# Global, regional, and national burden of ischemic heart disease attributable to environmental risk factors from 1990 to 2021: a systematic analysis based on the 2021 global burden of disease study

**DOI:** 10.3389/fpubh.2025.1630811

**Published:** 2025-12-10

**Authors:** Hongtao Huang, Qingpiao Sun, Wenqing Lv, Hanjun Zhao, Yu Huang

**Affiliations:** 1 Department of Cardiology, Gongli Hospital of Shanghai Pudong New Area, Shanghai, China; 2 Department of Cardiology, Shanghai Tenth People’s Hospital, Tongji University School of Medicine, Shanghai, China; 3 Department of Cardiology, Shuguang Hospital Affiliated to Shanghai University of Traditional Chinese Medicine, Shanghai, China

**Keywords:** ischemic heart disease, socio-demographic index, health inequality, global burden of disease 2021, Bayesian age-period-cohort model

## Abstract

**Background:**

Ischemic heart disease (IHD) attributable to environmental factors is a global public health challenge. This study assesses the global burden of IHD due to environmental factors from 1990 to 2021 using Global Burden of Disease (GBD) 2021 data and projects trends to 2051.

**Methods:**

Data on the mortality and disability-adjusted life years (DALYs) rates of IHD attributable to environmental factors were extracted from GBD 2021. The age-period-cohort (APC) model was employed to assess the independent effects of age, period, and cohort on the burden of IHD related to environmental factors. Additionally, the Bayesian age-period-cohort (BAPC) model was utilized to project future trends in the disease burden of IHD through 2051.

**Results:**

In 2021, age-standardized mortality rate (ASMR) and age-standardized DALYs rate (ASDR) for IHD due to environmental factors were 39.70 (95% UI: 30.74, 47.81) and 827.52 (95% UI: 648.13, 987.15) per 100,000 population, respectively. Socio-demographic Index (SDI) was negatively correlated with ASMR and ASDR. APC analysis showed declining trends, while BAPC predicts ASMR and ASDR will decline by 53.67 (95% UI: 11.48, 95.86) and 986.76 (95% UI: 291.27, 1682.25) per 100,000 population by 2051.

**Conclusion:**

The number of mortality and DALYs associated with IHD due to environmental factors has exhibited an increasing trend globally. However, projections suggest a general decline in the ASMR and ASDR of IHD burden attributable to environmental factors by 2050. Ambient particulate matter and household air pollution are the predominant contributors to the global IHD burden.

## 1 Introduction

Ischemic Heart Disease (IHD) is a chronic condition characterized by reduced blood supply to the coronary arteries, leading to myocardial damage (1, 2). As one of the leading causes of mortality among non-communicable diseases (NCDs) worldwide, IHD poses a significant public health challenge, contributing to a substantial global medical and economic burden (3, 4). In response to the growing impact of NCDs, the United Nations has set a global target to reduce premature mortality from NCDs by one-third among individuals aged 30 to 70 years by 2030 (5). In 2019, IHD accounted for nearly 200 million years of life lost (YLLs) due to premature mortality, underscoring the urgent need for effective prevention and control strategies to mitigate this global health burden (6). Furthermore, IHD ranked as the second leading contributor to disability-adjusted life years (DALYs) across all age groups, accounting for 7.2% of total DALYs worldwide. Its burden was particularly pronounced among individuals aged 50, 74 years and those aged 75 years and older, making it the leading cause of mortality across all regions (1). The substantial healthcare costs and service demands associated with IHD further highlight the necessity of early interventions to prevent complications and reduce disease burden (7). According to projections from the Global Burden of Disease (GBD) 2019 study, both the prevalence and mortality of IHD were expected to rise significantly in the coming decades (1, 8).

The etiology of IHD is multifactorial, involving genetic, environmental, and behavioral determinants (6). Among these, environmental factors, particularly air pollution, have been increasingly recognized as significant contributors to IHD risk (9). Recent evidence confirms that both ambient and household air pollution act as major environmental drivers of IHD mortality, with their combined impact being particularly severe in low- and middle-income countries (10). Air pollution is the fourth leading global risk factor for morbidity and mortality, with more than 50% of air pollution-related mortality attributable to IHD and stroke (11, 12). Notably, PM2.5 pollution has been shown to disproportionately exacerbate the burden of environmentally induced IHD in low- and middle-income countries, particularly in regions such as Asia, Oceania, and sub-Saharan Africa (13). Critically, the burden of environmentally driven IHD is profoundly shaped by socio-economic development levels, which influence both exposure to pollutants and capacity for healthcare response (14).

However, existing studies examining the association between environmental risk factors and IHD often suffer from regional limitations or focus on individual risk factors, failing to comprehensively elucidate the long-term impact of environmental exposures on disease burden. Furthermore, a critical gap persists in understanding how socio-economic development (period effects), population aging (age effects), and generational exposure histories (cohort effects) interact to shape the temporal trends of environmentally attributable IHD. Consequently, robust, model-based long-term projections of this specific burden, which are crucial for proactive public health planning, remain scarce. Therefore, a multidimensional analysis of the relationship between environmental risk factors and health outcomes is essential to inform effective prevention and intervention strategies.

To address these gaps, we conducted a comprehensive assessment of the burden of IHD attributable to environmental factors from 1990 to 2021, leveraging the most recent data from the GBD 2021 study. Our study had three primary objectives: (1) to quantify the contribution of environmental factors to the global burden of IHD, (2) to analyze the independent effects of age, period, and cohort on the burden of environmentally induced IHD using the age-period-cohort (APC) model, and (3) to project trends in IHD burden attributable to environmental factors over the next 30 years. The findings of this study aim to provide a scientific basis for policymakers and public health practitioners to develop targeted prevention strategies and implement evidence-based interventions to mitigate the future burden of IHD.

## 2 Methods

### 2.1 Data source

This study utilized data from the GBD 2021, published by the Institute for Health Metrics and Evaluation (IHME).^1^ The GBD database is the most comprehensive and detailed source of information on global diseases, injuries, and risk factors. The classification of world regions and locations followed the standard geographic hierarchy used in the GBD 2021 study, which includes 204 countries and territories aggregated into 21 regional groups. The complete list of locations and their regional assignments is available in the primary GBD 2021 publications (15). All data used in this study are publicly available and do not require additional ethical approval (16).

### 2.2 Case definition and data standards

Cases of IHD were defined according to the International Classification of Diseases, Tenth Revision (ICD-10) codes I20-I25 (17). Mortality and prevalence data in the GBD 2021 study were mapped to these codes. The Cause of Death Ensemble model (CODEm), a robust modeling framework developed by IHME, was used as the standard for calculating mortality estimates. This framework systematically incorporates and weights data from vital registration systems, verbal autopsy, and other sources to produce cause-specific mortality estimates (18).

### 2.3 Data collection

The GBD study employs DisMod-MR 2.1, a Bayesian meta-regression modeling framework, to estimate disease burden by integrating multiple data sources. This model accounts for measurement differences in mortality rates, case definitions, and data collection methodologies across different studies, allowing for standardized estimates of IHD mortality attributable to environmental factors (19).

Disability-adjusted life years (DALYs) rate is an indicator for measuring the disease burden, which combines the years of life lost (YLLs) due to premature death and the years of life lost due to disability (YLDs) caused by diseases. This indicator enables a comprehensive assessment of the impact of diseases and medical interventions, which is particularly important for disease prevention and control. The computation of DALYs follows the standard GBD methodology, where DALYs are the sum of YLLs and YLDs, providing a comprehensive metric of overall health loss (20). Using the relevant data provided by GBD 2021, we calculated the mortality and DALYs rates for diseases attributed to environmental factors and further analyzed their trends (21).

### 2.4 Socio-demographic index

The Socio-demographic index (SDI) is a composite measure that reflects the social and economic conditions influencing health outcomes. It is calculated based on educational attainment, per capita income, and total fertility rate, ranging from 0 to 1, where higher values indicate greater socio-economic development. Based on SDI values, countries and regions are classified into five categories: low SDI [0–0.4658), low-middle SDI [0.4658–0.6188), middle SDI [0.6188–0.7120), high-middle SDI [0.7120–0.8103), and high SDI [0.8103–1.0000] (22). This study analyzed the association between the burden of IHD attributable to environmental factors and socio-economic development levels across different SDI regions (23).

### 2.5 APC model

To assess the independent effects of age, period, and birth cohort on IHD burden, we applied the APC model, which provides a robust framework for disentangling the contributions of biological aging, historical period effects, and generational influences on disease trends. This model is particularly suited for identifying the underlying drivers of long-term trends in disease burden, as demonstrated in prior GBD-based studies (24). This approach goes beyond traditional epidemiological methods by incorporating the effects of social and technological changes over time. In our study, it allows us to determine whether trends in environmentally attributable IHD are driven by population aging, period-specific interventions, or the cumulative exposures of specific birth cohorts. The input data for the APC model included mortality and DALYs rates estimates for IHD attributable to environmental factors, as well as population data for each country and region from GBD 2021. The APC model requires equal intervals for both age groups and periods, ensuring consistency in the analysis (i.e., 5-year age groups aligned with 5-year periods) (25).

### 2.6 Health inequality analysis

To evaluate inequalities in the burden of attributable to environmental factors across socio-economic groups, we utilized two standard epidemiological indicators. The slope index of inequality (SII), a measure of absolute inequality, was calculated by regressing national mortality and DALYs rates on a ranked SDI scale, capturing the gradient of disparities in disease burden across SDI levels. The concentration index (CI), a measure of relative inequality, was derived from the Lorenz curve, quantifying the deviation of actual health outcomes from perfect equality. CI was computed by integrating cumulative mortality and DALYs rates against the cumulative proportion of the population ranked by SDI, with higher CI values indicating a more unequal distribution of disease burden (26).

### 2.7 Risk factors

In addition to analyzing overall IHD burden, this study also examined the impact of specific environmental risk factors on IHD mortality and DALYs rates. According to GBD 2021, key environmental risk factors include: household air pollution, ambient particulate matter pollution (PM2.5 exposure), low temperature, high temperature and lead exposure. The estimation of risk-attributable burden was based on the exposure distributions, theoretical minimum risk exposure levels (TMRELs), and effect sizes defined within the GBD 2021 comparative risk assessment framework (27). We assessed the relative contribution of each risk factor to the burden of IHD, providing insights into potential intervention strategies.

### 2.8 Predictions of the burden

To predict future trends in the burden of IHD attributable to environmental factors, we applied the Bayesian age-period-cohort (BAPC) model, a statistical approach that leverages historical trends to estimate future disease burden. The BAPC model is well-established for generating stable and interpretable long-term forecasts of disease burden, as evidenced by its application in forecasting other non-communicable diseases (28). The Integrated Nested Laplace Approximation (INLA) method was used to improve computational efficiency and ensure the accuracy of long-term predictions. This approach is critical for projecting the future course of IHD burden under current trends, thereby informing long-term public health planning. This model was used to forecast mortality and DALYs rates of IHD attributable to environmental factors up to 2051 (27).

### 2.9 Statistical analysis

To assess temporal trends in the age-standardized rates (ASR) of IHD attributable to environmental factors, we estimated the annual percentage change (EAPC). The ASR per 100,000 population was calculated using the following formula (29):


$$\mathrm{ASR}=\frac{\sum_{i=1}^{A}a_{i}w_{i}}{w_{i}}\times 100000$$

where *a_i_* represents the age-specific population proportion, and *W_i_*: the number of people in the corresponding i^th^ age group in the standard population, A: the number of age groups.

The EAPC is calculated based on a log-linear regression model, where time is considered an independent variable. By fitting the natural logarithm of ASR to a straight line, the trend over time can be quantified using the slope of this regression line. The EAPC is computed using the following formula (30):


y=α+βx+ε

EAPC=100×[exp(β)−1]

where *x*: year, *y*: the natural logarithm of the rate, *α*: intercept, *β*: slope, *ε*: random error. The 95% uncertainty interval (UI) of EAPC also comes from this fitted model. Data analysis was conducted using R software (version 4.4.1). To ensure reproducibility, the R code used for analysis has been deposited in a permanent repository. The code is available via Zenodo at the following Digital Object Identifier (DOI): [10.6084/m9.figshare.30549986]. Figures were refined using Adobe Illustrator (version 2024).

## 3 Results

### 3.1 Global trends of IHD

Between 1990 and 2021, the ASMR for IHD attributable to environmental factors declined from 57.64 per 100,000 population (95% UI: 45.79, 68.89) to 39.70 per 100,000 population (95% UI: 30.74, 47.81), with an EAPC of −1.27 (95% UI: −1.34, −1.19; Table 1). Similarly, the ASDR decreased from 1179.60 per 100,000 population (95% UI: 942.76, 1410.91) in 1990 to 827.52 per 100,000 population (95% UI: 648.13, 987.15) in 2021, with an EAPC of −1.23 (95% UI: −1.31, −1.16; Table 1).

**Table 1:** Mortality and ASMR for IHD due to environmental factors in 1990 and 2021, with EAPCs from 1990 to 2021.

Location	Number in 1990 (95%CI)	Age-standardized mortality rate per 100,000 population in 1990 (95%UI)	Number in 2021 (95%CI)	Age-standardized mortality rate per 100,000 population in 2021 (95%UI)	1990–2021 EAPC (95%CI)
Global	1,994,917 (1,595,529,2,389,340)	57.64 (45.79,68.89)	3,302,951 (2,562,529,3,977,698)	39.7 (30.74,47.81)	−1.27 (−1.34, −1.19)
High SDI	448,498 (328,846,572,395)	40.74 (29.82,51.97)	283,197 (207,585,355,256)	12.06 (8.84,15.01)	−4.24 (−4.32, −4.15)
High-middle SDI	570,481 (441,037,704,233)	68.51 (52.16,84.43)	751,299 (576,094,924,686)	39.08 (29.91,48.03)	−2.08 (−2.32, −1.84)
Middle SDI	469,053 (380,961,556,424)	57.17 (45.91,67.78)	1,133,319 (865,376,1,381,260)	48 (36.18,58.25)	−0.44 (−0.58, −0.29)
Low-middle SDI	381,876 (310,676,451,884)	71.42 (58.02,84.41)	872,505 (694,815,1,031,237)	67.64 (53.54,80.26)	−0.03 (−0.12,0.06)
Low SDI	121,809 (96,643,145,923)	63.93 (51.08,76.53)	259,745 (207,978,309,125)	61.93 (49.91,73.6)	0.01 (−0.11,0.13)
Andean Latin America	8,152 (6,461,9,902)	44.7 (35.26,54.22)	10,696 (7,485,14,433)	18.92 (13.23,25.51)	−3.2 (−3.49, −2.91)
Australasia	6,990 (3,514,11,079)	30.76 (15.42,48.75)	5,304 (3,278,7,235)	8.59 (5.36,11.67)	−4.55 (−4.74, −4.37)
Caribbean	13,823 (8,870,19,296)	57.97 (37.04,81.19)	19,180 (12,226,26,468)	35.22 (22.52,48.62)	−1.58 (−1.77, −1.38)
Central Asia	48,062 (34,916,62,842)	117.14 (84.99,153.26)	63,357 (48,386,76,915)	96.01 (73.21,116.52)	−1.1 (−1.41, −0.79)
Central Europe	133,350 (92,488,171,385)	99.85 (68.89,128.32)	88,799 (67,619,109,407)	37.47 (28.57,46.13)	−3.55 (−3.73, −3.38)
Central Latin America	34,422 (24,983,43,673)	49.01 (35.55,62.23)	67,628 (44,626,91,092)	28.5 (18.77,38.43)	−1.92 (−2.07, −1.77)
Central Sub-Saharan Africa	11,409 (8,363,15,223)	66.07 (49.75,85.88)	22,767 (16,285,30,273)	54.88 (39.13,72.96)	−0.8 (−0.87, −0.73)
East Asia	295,511 (233,070,358,054)	48.67 (38.8,58.36)	894,463 (669,150,1,129,102)	48.42 (36.07,60.91)	0.44 (0.05,0.84)
Eastern Europe	251,788 (162,574,335,861)	103.55 (66.6,138.4)	189,276 (136,310,247,483)	52.92 (38.12,69.22)	−2.9 (−3.4, −2.38)
Eastern Sub-Saharan Africa	22,768 (18,147,27,759)	36.36 (28.83,44.09)	50,766 (39,128,61,291)	36.86 (28.51,44.69)	−0.14 (−0.23, −0.05)
High-income Asia Pacific	27,630 (15,976,42,388)	15.67 (9.03,24.07)	35,912 (24,578,47,050)	6.01 (4.22,7.76)	−3.11 (−3.31, −2.91)
High-income North America	144,974 (90,721,206,936)	39.95 (24.99,57.06)	76,918 (49,741,104,284)	10.86 (7.11,14.65)	−4.69 (−4.93, −4.46)
North Africa and Middle East	169,398 (133,888,203,091)	120.49 (94.5,144.48)	330,659 (260,073,405,105)	86.56 (67.35,105.68)	−1.1 (−1.13, −1.08)
Oceania	1927 (1,435,2,561)	77.05 (58.29,101.72)	4,423 (3,186,5,786)	67.34 (49.04,87)	−0.39 (−0.46, −0.33)
South Asia	373,446 (301,568,448,915)	72.42 (58.4,86.69)	986,089 (778,522,1,171,577)	74.11 (58.71,88.22)	0.24 (0.14,0.35)
Southeast Asia	113,560 (89,415,139,254)	51.85 (40.91,63.79)	215,368 (158,594,270,525)	37.7 (27.76,47.09)	−1.11 (−1.28, −0.93)
Southern Latin America	18,733 (12,666,25,326)	44.52 (29.98,60.24)	11,943 (8,401,15,764)	13.23 (9.31,17.44)	−3.69 (−3.88, −3.49)
Southern Sub-Saharan Africa	7,140 (5,511,8,813)	30.3 (23.12,37.58)	13,712 (10,436,16,961)	28.48 (21.65,35.15)	−0.24 (−0.68,0.2)
Tropical Latin America	33,243 (22,074,45,587)	42.29 (27.92,57.85)	34,882 (21,394,47,427)	13.89 (8.51,18.93)	−3.57 (−3.65, −3.48)
Western Europe	240,865 (158,124,335,087)	40.58 (26.52,56.41)	102,627 (72,752,132,254)	8.87 (6.4,11.36)	−5.22 (−5.37, −5.06)
Western Sub-Saharan Africa	37,726 (29,037,48,313)	52.95 (41.13,67.8)	78,182 (59,816,95,998)	51.54 (39.15,63.13)	−0.08 (−0.22,0.06)

Among SDI regions, the low-middle SDI group exhibited the highest ASMR (67.64 per 100,000 population, 95% UI: 53.54–80.26) and ASDR (1482.02 per 100,000 population, 95% UI: 1181.93–1753.75), with EAPCs of −0.03 (95% UI: −0.12. 0.06) and −0.19 (95% UI: −0.27, −0.11), respectively. In contrast, the high SDI group recorded the lowest ASMR (12.06 per 100,000 population, 95% UI: 8.84–15.01) and ASDR (240.11 per 100,000 population, 95% UI: 182.12–296.64), with EAPCs of −4.24 (95% UI: −4.32, −4.15) and −4.00 (95% UI: −4.09, −3.92), respectively (Table 1).

Regionally, Central Asia had the highest ASMR (96.01 per 100,000 population, 95% UI: 73.21–116.52) and ASDR (1749.56 per 100,000 population, 95% UI: 1328.65–2124.50), with EAPCs of −1.10 (95% UI: −1.41, −0.79) and −1.38 (95% UI: −1.74, −1.02), respectively. Conversely, the High-income Asia Pacific region exhibited the lowest ASMR (6.01 per 100,000 population, 95% UI: 4.22–7.76) and ASDR (113.84 per 100,000 population, 95% UI: 82.47–145.82), with EAPCs of −3.11 (95% UI: −3.31, −2.91) and −2.90 (95% UI: −3.03, −2.77), respectively (Table 1).

**Figure 1:**
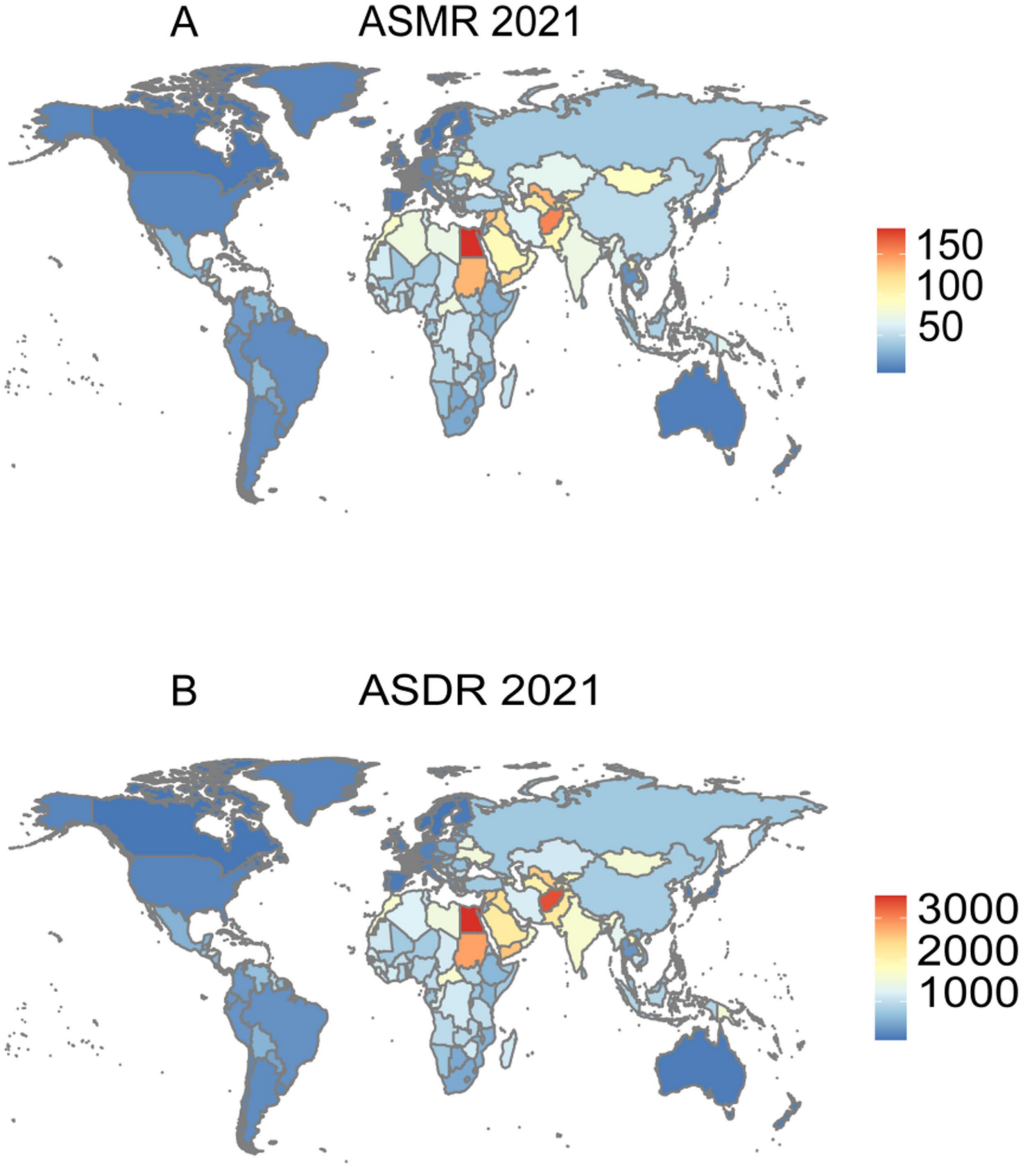
Burden of IHD attributable to environmental factors across 204 countries and regions in 2021. **(A)** ASMR of IHD due to environmental factors. **(B)** ASDR of IHD due to environmental factors.

At the national level, Egypt (181.22 per 100,000, 95% UI: 139.82–227.91), Afghanistan (154.48 per 100,000, 95% UI: 113.30–201.29), and Vanuatu (141.56 per 100,000, 95% UI: 108.04–176.99) recorded the highest ASMRs in 2021. Conversely, the lowest ASMRs were observed in Puerto Rico (4.08 per 100,000, 95% UI: 1.34–6.83), San Marino (4.47 per 100,000, 95% UI: 2.72–6.59), and Norway (5.07 per 100,000, 95% UI: 3.26–6.86; Figure 1A). Similarly, the highest ASDRs were reported in Egypt (3538.05 per 100,000, 95% UI: 2708.97–4480.72), Afghanistan (3361.53 per 100,000, 95% UI: 2399.57–4478.56), and Vanuatu (3265.58 per 100,000, 95% UI: 2457.72–4084.33). In contrast, the lowest ASDRs were observed in San Marino (78.89 per 100,000, 95% UI: 48.91–116.05), Puerto Rico (82.50 per 100,000, 95% UI: 28.45–139.64), and Norway (83.19 per 100,000, 95% UI: 54.37–112.99; Figure 1B).

### 3.2 Trends of IHD due to environmental factors by age and sex

**Figure 2:**
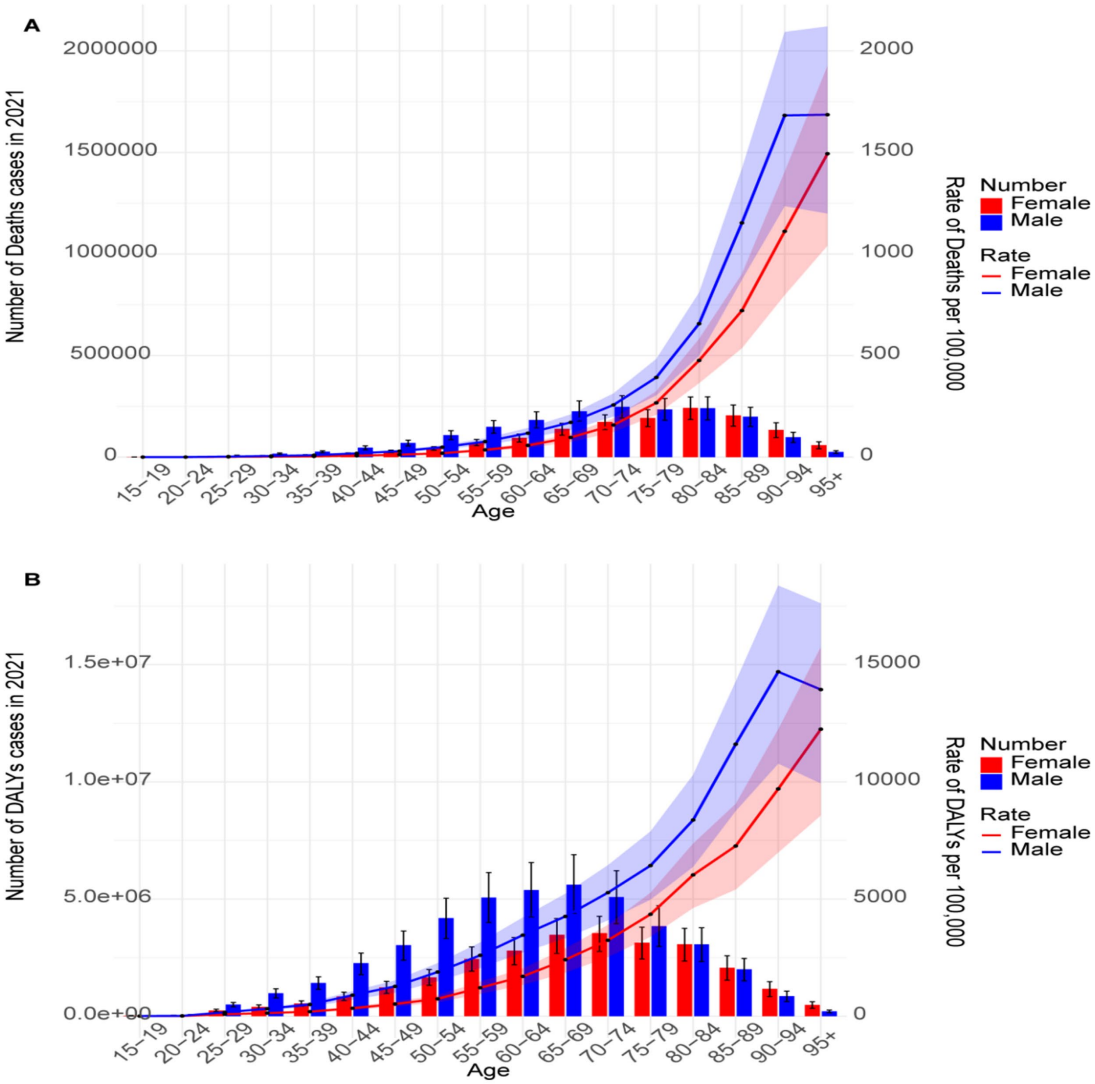
Global burden of IHD due to environmental factors stratified by age and sex in 2021. **(A)** Global mortality and mortality rates by age and sex. **(B)** DALYs and DALYs rates by age and sex.

The mortality rate and number of mortality due to IHD increased with age, with higher mortality rates in males compared to females across all age groups. The highest mortality in males was observed in the 70–74 age group, while the highest female mortality was recorded in the 80–84 age group (Figure 2A). Similarly, DALYs and DALYs rates also increased with age, with males experiencing a higher DALYs burden than females in most age groups. The highest number of DALYs in males was observed in the 65–69 age group, whereas for females, the peak was in the 70–74 age group (Figure 2B).

### 3.3 Trends in environmental factors leading to IHD by SDI classification

**Figure 3:**
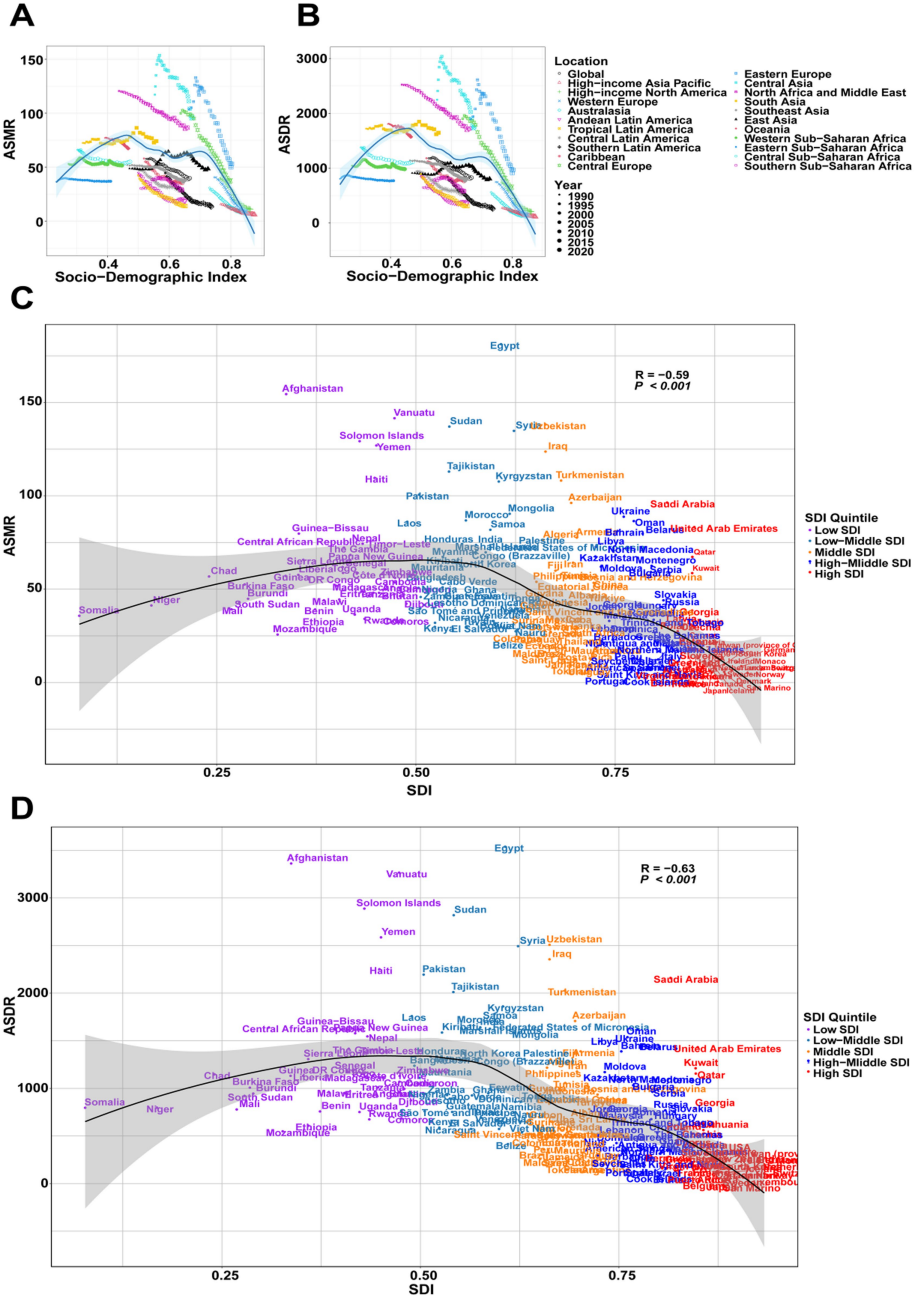
Trends in environmental factors leading to IHD from 1990 to 2021 by SDI classification. **(A)** Relationship between IHD ASMR and SDI across 21 GBD regions. **(B)** Correlation analysis of IHD ASMR and SDI. **(C)** Relationship between IHD ASDR and SDI across 21 GBD regions. **(D)** Correlation analysis of IHD ASDR and SDI.

In 2021, both the ASMR and ASDR exhibited a wave-like relationship with the SDI. When SDI was below 0.46, both ASMR and ASDR increased rapidly. Between SDI 0.46 and 0.6, these rates gradually declined, followed by a slow increase in the SDI range of 0.6 to 0.7. However, when SDI exceeded 0.7, ASMR and ASDR sharply decreased (Figures 3A,C). While this trend was generally consistent across regions, Sub-Saharan Africa, Central Asia, Central Europe, and Eastern Europe exhibited higher overall levels of ASMR and ASDR (Figures 3A,C). Further analysis indicated that the burden of IHD attributable to environmental factors was most pronounced in middle SDI regions, with ASMR and ASDR peaking around an SDI of 0.6 before gradually declining (Figures 3B,D). Overall, SDI demonstrated a negative correlation with both ASMR and ASDR, suggesting that the burden of IHD was highest in middle SDI regions and relatively lower in high SDI countries.

### 3.4 APC model

**Figure 4:**
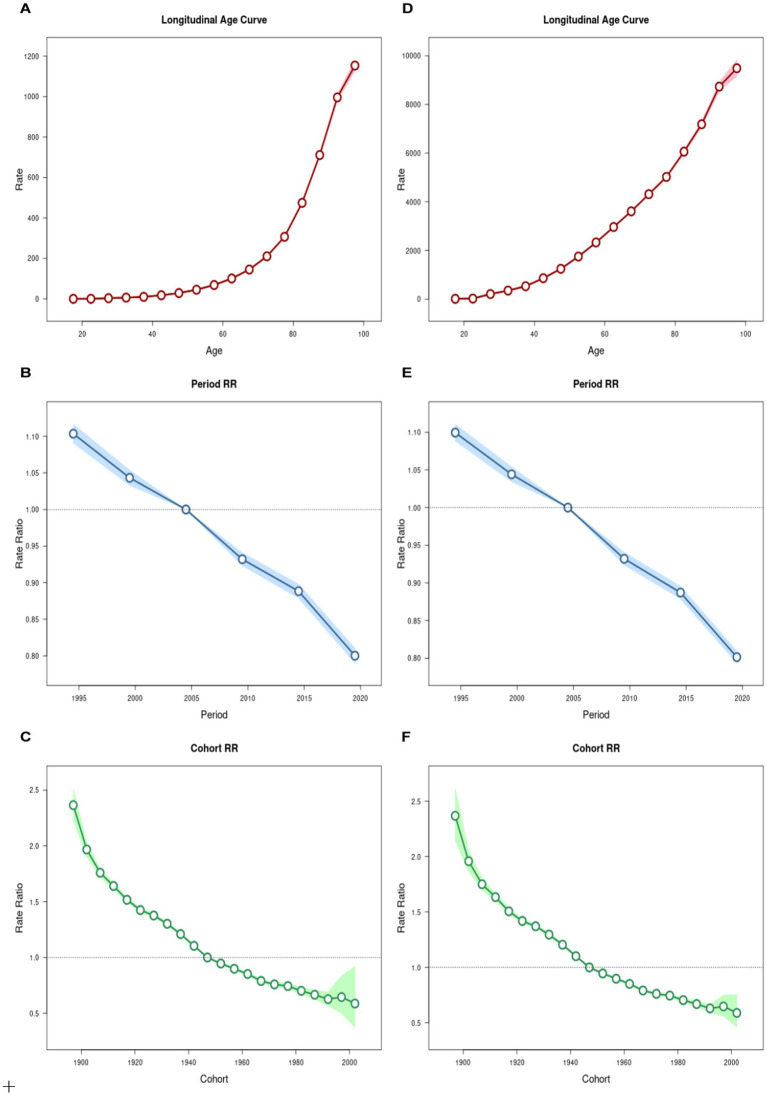
Age-period-cohort analysis of the global mortality and DALYs rate for IHD due to Environmental Factors. **(A)** Age effect on mortality rate; **(B)** period effect on mortality rate; **(C)** cohort effect on mortality rate; **(D)** age effect on DALYs rate; **(E)** period effect on DALYs rate; **(F)** cohort effect on DALYs rate.

Using the APC model, this study assessed the burden of IHD attributable to environmental factors. The results indicated an increase in global mortality and DALYs rates of IHD with advancing age, reaching their highest levels in the 95 + age group, at 1153.58 (95% UI: 1126.51, 1181.31) per 100,000 population and 9485.96 (95% UI: 9156.76, 9827.01) per 100,000 population (Figures 4A,D), respectively. Period effects demonstrated a declining trend in both mortality and DALYs rates. Between 2017 and 2021, the risks ratios (RR) of mortality and DALYs were at their lowest, recorded at 0.8 (95% UI: 0.79, 0.81) and 0.802 (95% UI: 0.794, 0.809; Figures 4B,E), respectively. Cohort effects revealed a downward trend in mortality and DALYs rates among more recent birth cohorts. Specifically, the cohort born between 2002 and 2006 exhibited the lowest rates, with RRs of 0.588 (95% UI: 0.375, 0.92) for mortality and 0.589 (95% UI: 0.459, 0.755) for DALYs rates (Figures 4C,F).

### 3.5 Health inequality analysis

**Figure 5:**
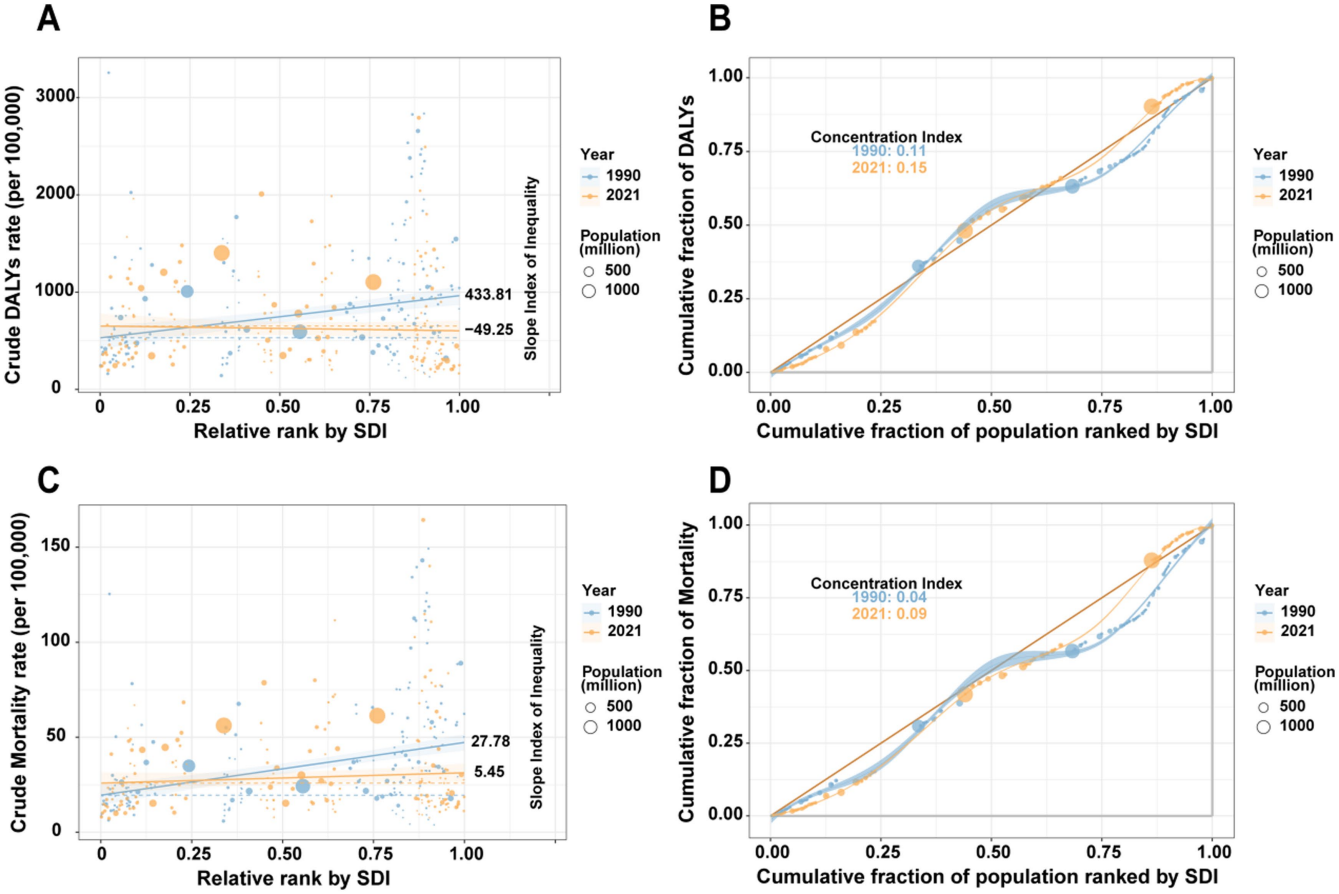
Absolute and relative cross-national inequalities in IHD mortality and DALYs rates from 1990 to 2021. **(A)** Health inequality regression curve for mortality rate. **(B)** Concentration curve for mortality rate. **(C)** Health inequality regression curve for DALYs rate. **(D)** Concentration curve for DALYs rate.

From 1990 to 2021, the health inequality outcomes of IHD due to environmental factors showed a downward trend in the SII for both mortality and DALYs rates, which decreased from 27.78 to 5.45 and from 433.81 to 49.25, respectively, indicating a reduction in the absolute disparity in mortality and DALYs rates (Figures 5A,C). However, the CI for mortality and DALYs rates increased, from 0.04 to 0.09 and from 0.11 to 0.15, respectively, suggesting a growing inequality in the distribution of these rates across different SDI levels (Figures 5B,D). Overall, while some indicators show a reduction in the absolute disparity of health inequalities, the overall unevenness in distribution has increased.

From 1990 to 2021, health inequality outcomes related to IHD due to environmental factors showed a downward trend in the SII for both mortality and DALYs rates. The SII for mortality decreased from 27.78 to 5.45, while the SII for DALYs declined from 433.81 to 49.25, indicating a reduction in absolute disparities in these measures (Figures 5A,C). However, the CI for both mortality and DALYs increased from 0.04 to 0.09 and from 0.11 to 0.15, respectively, suggesting a growing inequality in the distribution of these rates across different SDI levels (Figures 5B,D). Taken together, these findings indicate that while absolute disparities in health inequalities have declined, relative inequalities in distribution have increased.

### 3.6 Risk factor analysis

**Figure 6:**
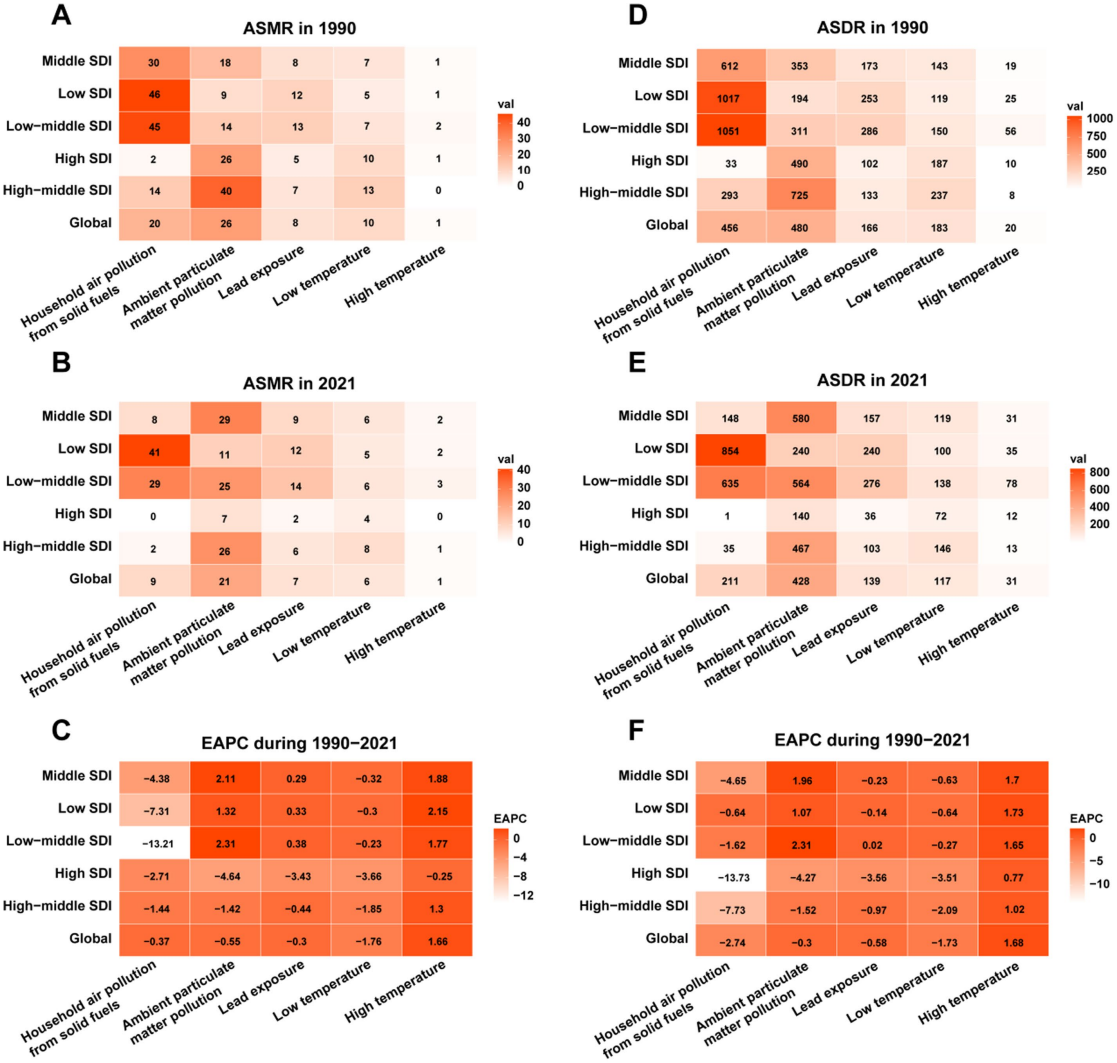
Risk factors for IHD caused by environmental factors in 1990 and 2021 globally and across 5 SDI regions. **(A)** ASMR in 1990, **(B)** ASMR in 2019, **(C)** EAPC of ASMR from 1990 to 2019, **(D)** ASDR in 1990, **(E)** Annual ASDR of IHD caused by environmental factors in 2019, **(F)** EAPC of ASDR from 1990 to 2019.

Between 1990 and 2021, ambient particulate matter pollution was the primary environmental risk factor contributing to IHD mortality and DALYs rates in global, high SDI, and high-middle SDI regions, although its contribution declined annually. Mortality rates decreased from 25.55 per 100,000 (95% UI: 17.27, 34.24) to 20.85 per 100,000 (95% UI: 14.63, 27.57) globally, from 26.12 per 100,000 (95% UI: 15.90, 36.69) to 6.71 per 100,000 (95% UI: 4.54, 9.00) in high SDI regions, and from 40.02 per 100,000 (95% UI: 25.87, 55.56) to 25.78 per 100,000 (95% UI: 17.84, 33.84) in high-middle SDI regions, with respective estimated annual percentage changes (EAPCs) of −0.55 (95% UI: −0.72, −0.38), −4.64 (95% UI: −4.73, −4.55), and −1.42 (95% UI: −1.59, −1.25; Figures 6A–C). Similarly, DALYs rates declined in these regions (Figures 6D–F).

In middle SDI, low-middle SDI, and low SDI regions, household air pollution from solid fuels was the leading environmental contributor to IHD mortality and DALYs rates, although its impact also exhibited a declining trend. Mortality rates decreased from 29.54 per 100,000 (95% UI: 21.71, 38.06) to 29.48 per 100,000 (95% UI: 19.55, 39.08) in middle SDI regions, from 45.37 per 100,000 (95% UI: 34.95, 56.20) to 28.85 per 100,000 (95% UI: 17.29, 42.01) in low-middle SDI regions, and from 45.78 per 100,000 (95% UI: 35.86, 56.90) to 40.63 per 100,000 (95% UI: 31.24, 50.09) in low SDI regions, with respective EAPC of 2.11 (95% UI: 1.9, 2.32), 2.31 (95% UI: 2.06, 2.55), and 1.32 (95% UI: 0.92, 1.72; Figures 6A–C). Similar trends were observed for DALYs rates (Figures 6D–F). These findings highlight that while the disease burden from ambient particulate matter pollution and household air pollution has decreased, they remain the primary environmental factors contributing to IHD.

### 3.7 Predictions of the burden of IHD

**Figure 7:**
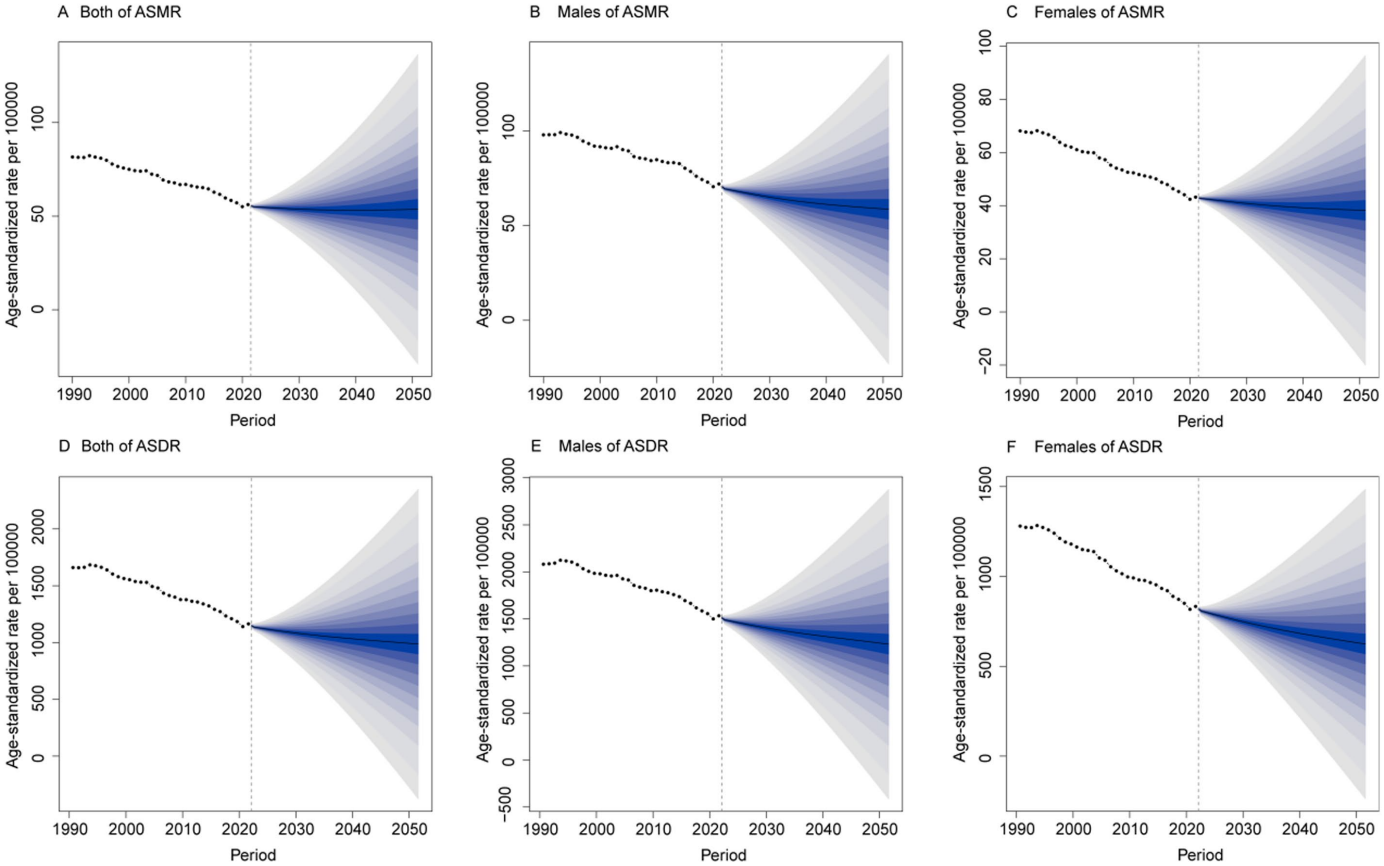
Prediction of IHD trends due to environmental factors over the next 30 years. **(A)** ASMR for both sexes. **(B)** ASMR for males. **(C)** ASMR for females. **(D)** ASDR for both sexes. **(E)** ASDR for males. **(F)** ASDR for females.

Based on the BAPC model, the burden of IHD attributable to environmental factors is projected to continue declining in terms of ASMR and ASDR by 2051. By 2051, the ASMR is expected to be 53.67 (95% UI: 11.48, 95.86) per 100,000 for both sexes, 58.65 (95% UI: 16.92, 100.38) per 100,000 for males, and 38.21 (95% UI: 8.48, 67.93) per 100,000 for females. Similarly, the ASDR is projected to reach 986.76 (95% UI: 291.27, 1682.25) per 100,000 for both sexes, 1230.79 (95% UI: 391.05, 2070.53) per 100,000 for males, and 624.08 (95% UI: 184.53, 1063.63) per 100,000 for females (Figure 7).

## 4 Discussion

This study provides a comprehensive assessment of the global, regional, and national burden of IHD attributable to environmental factors from 1990 to 2021, with projections extending to 2051. The findings underscore the substantial and persistent impact of environmental risk factors on IHD, revealing notable disparities across socioeconomic and geographic contexts. These global patterns were reflected in substantial heterogeneities at finer geographical resolutions.

At the national level, countries with the highest burden included Egypt, Afghanistan, and Vanuatu. These nations share common challenges including limited healthcare infrastructure, high exposure to household and ambient air pollution, and ongoing environmental degradation. In middle SDI regions, rapid industrialization coupled with inadequate environmental regulations has created particularly severe exposure scenarios, explaining their disproportionately high ASMR and ASDR indicators. The primary environmental risk factors contributing to the global IHD burden in 2021 were ambient particulate matter pollution (PM2.5), household air pollution, and exposure to extreme temperatures. These factors are well-established contributors to cardiovascular diseases, including IHD, due to their adverse effects on both the respiratory and cardiovascular systems. Among these, ambient PM2.5 exposure emerged as the predominant contributor, particularly in low- and middle-income regions where elevated exposure levels result from reliance on biomass fuels and suboptimal air quality. This finding is consistent with previous studies identifying air pollution as a major environmental determinant of cardiovascular disease (2, 31, 32). While the direct contribution of air pollution to IHD is well-documented, emerging evidence suggests that other environmental factors, such as extreme temperatures, are increasingly significant contributors to the disease burden. Both extreme heat and cold have been associated with exacerbations of cardiovascular conditions, leading to elevated IHD mortality, particularly among older populations (33). Additionally, household air pollution, largely driven by solid fuel combustion, remains a critical public health concern in low-income countries where access to clean energy alternatives is limited. Recent studies highlight that household air pollution substantially contributes to the IHD burden in regions such as South Asia, emphasizing the urgent need for improved access to cleaner cooking technologies (34). These findings reinforce the necessity of mitigating environmental risk factors to reduce the global burden of IHD.

The divergent trends observed in the health inequality indices, a declining SII alongside a rising CI, reveal a nuanced picture of how the environmental IHD burden is distributed globally. This pattern suggests that while broad, absolute gains in reducing the IHD burden have been made (as reflected in the SII), these gains have not been equitably shared. The increasing CI indicates a widening relative disadvantage, meaning the burden is becoming progressively more concentrated among socio-economically disadvantaged populations over time. This could be driven by slower adoption of environmental regulations, persistent reliance on polluting fuels, and lagging healthcare infrastructure in lower-SDI regions, even as higher-SDI regions accelerate their improvements. Therefore, the overall decline in burden masks a critical public health challenge: the escalation of relative health inequity related to environmental risks.

The analysis revealed significant disparities in the IHD burden across different age groups, sexes, and socioeconomic levels. The burden attributable to environmental factors increases with age, particularly among individuals aged 50 and above, reflecting the cumulative impact of long-term exposure to environmental pollutants. This pattern aligns with well-established risk factors, such as hypertension, diabetes, and dyslipidemia, which become more prevalent with aging (35). Males exhibited higher mortality and DALYs rates than females, with peak mortality observed at ages 70–74 for males and 80–84 for females. These findings are consistent with existing literature suggesting that biological differences, lifestyle factors, and occupational exposures contribute to an increased IHD risk in males (6). Furthermore, an inverse correlation between IHD burden and SDI was observed, with middle-SDI regions experiencing the highest burden. This underscores the complex interplay between socioeconomic development and environmental health risks, wherein rapid industrialization and urbanization in middle-SDI regions exacerbate exposure to environmental pollutants (31, 32). Sex-based differences in environmental exposure also play a crucial role. In low- and middle-income countries, males are more likely to be exposed to outdoor air pollution and occupational hazards, which may explain their higher IHD burden (36). Conversely, females in these settings face greater exposure to household air pollution due to traditional cooking methods, making household air pollution a significant risk factor for them (37, 38). These gender-specific exposure patterns highlight the need for targeted interventions addressing distinct environmental health risks faced by each sex.

The study found that the burden of IHD attributable to environmental factors was highest in low-middle SDI regions and lowest in high SDI regions. This finding supports the notion that socioeconomic development is a key determinant in reducing the burden of environmentally induced diseases. High SDI countries generally implement stringent air quality regulations, provide better healthcare access, and allocate greater resources to mitigate environmental risk factors, leading to a lower IHD burden. In contrast, low- and middle-SDI regions, characterized by rapid industrialization and urbanization, experience higher air pollution exposure and possess limited healthcare infrastructure to address the growing IHD burden (31, 39, 40). The wave-like relationship between SDI and IHD burden, particularly the peak in ASMR and ASDR observed in regions with an SDI around 0.6, highlights a critical challenge for middle-income countries. These regions face a dual burden of environmental risk factors and an increasing prevalence of non-communicable diseases like IHD, yet their health systems remain insufficiently equipped to address prevention and treatment needs. In regions such as Central Asia and Sub-Saharan Africa, where environmental pollution and IHD prevalence are high, integrated public health policies that promote both environmental improvements and cardiovascular health interventions are urgently needed (9, 41).

The APC analysis revealed significant cohort effects, with successive birth cohorts exhibiting progressively lower IHD risk. The cohort born in 2002–2006 showed the lowest risk ratios, suggesting that younger generations benefit from cumulative improvements in environmental regulations, public health interventions, and living conditions over past decades (42). However, this protective cohort effect appears attenuated in regions with ongoing environmental degradation, emphasizing the need for sustained environmental policies.

Projections based on the BAPC model suggest a decline in both ASMR and ASDR for IHD attributable to environmental factors by 2051. However, significant regional variations are anticipated. These differential trajectories highlight the need for region-specific intervention strategies. This anticipated decline is likely driven by improvements in air quality, healthcare infrastructure, and the effectiveness of public health interventions targeting environmental risks. However, these projections are contingent upon sustained global efforts to combat air pollution, enhance environmental regulations, and implement targeted interventions in high-burden regions (43). Despite the projected decrease in the environmental burden of IHD, it will remain a major public health concern, particularly in low- and middle-income countries. Strengthening environmental policies, increasing investment in clean energy solutions, and raising public awareness about the health risks of environmental exposure will be essential (44–46). Furthermore, addressing the dual burden of infectious and non-communicable diseases in these regions will be critical for mitigating the future impact of IHD (41, 44, 47).

This study has several strengths, including comprehensive global coverage of 204 countries, use of advanced statistical models, integration of health inequality metrics, and provision of long-term projections to 2051. These features provide novel insights into the temporal evolution and equitable distribution of the environmental IHD burden.

However, the findings should be interpreted in the context of several limitations. Most importantly, the ecological nature of the GBD data limits causal inference at the individual level. Although the estimated associations are strong and biologically plausible, they cannot definitively establish causality. Other limitations include the potential for residual confounding from unmeasured variables (e.g., diet, physical activity) and the assumption in our projections that historical trends will continue, which may not account for future policy or technological changes. Furthermore, by focusing on environmental risk factors, our study did not fully account for other IHD determinants, such as genetic predisposition. Future research should explore the interplay between environmental and non-environmental risk factors to provide a more comprehensive understanding of the global IHD burden.

## 5 Conclusion

This study utilized data from the GBD 2021 to systematically assess the global, regional, and national burden of IHD attributable to environmental risk factors from 1990 to 2021. The findings indicate that while the absolute number of global deaths and DALYs due to IHD from environmental factors increased substantially by 65.57 and 54.3%, respectively, the age-standardized rates exhibited a declining trend, with ASMR and ASDR falling by 31.12 and 29.8%, respectively. This suggests that the rise in absolute numbers is likely driven by population growth and aging, while the decline in standardized rates reflects improvements in healthcare and environmental policies. The regional and SDI-level analysis showed that the highest ASMR and ASDR were observed in low-middle SDI regions and Central Asia. Analysis by age and sex revealed that the burden of IHD was more pronounced in males, with mortality peaking at ages 70–74 for men and 80–84 for women. Additionally, the inverse correlation between SDI and both ASMR and ASDR highlights a disproportionately higher burden in middle-SDI regions and a relatively lower burden in high SDI countries. Ambient particulate matter pollution and household air pollution were identified as the leading environmental contributors to the global IHD burden. To effectively mitigate this burden, countries must strengthen prevention strategies, enhance early screening efforts, and implement targeted interventions for IHD attributable to environmental risk factors. Addressing these factors is crucial for reducing future disease burdens and improving global public health outcomes.

## Data Availability

The original contributions presented in the study are included in the article/supplementary material, further inquiries can be directed to the corresponding author.

## Footnotes

1
https://vizhub.healthdata.org/gbd

## References

[1] Guan C Wu S Xu W Zhang J. Global, regional, and national burden of ischaemic heart disease and its trends, 1990-2019. Public Health. (2023) 223:57–66. doi: 10.1016/j.puhe.2023.07.010, 37604031

[2] Roth GA Mensah GA Johnson CO Global Burden of Cardiovascular Diseases Writing Group. Global burden of cardiovascular diseases and risk factors, 1990-2019: update from the GBD 2019 study. J Am Coll Cardiol. (2020) 76:2982–3021. doi: 10.1016/j.jacc.2020.11.010 33309175 PMC7755038

[3] India State-Level Disease Burden Initiative CVD Collaborators. The changing patterns of cardiovascular diseases and their risk factors in the states of India: the global burden of disease study 1990-2016. Lancet Glob Health. (2018) 6:e1339–51. doi: 10.1016/S2214-109X(18)30407-8, 30219317 PMC6227386

[4] GBD 2019 Diseases and Injuries Collaborators. Global burden of 369 diseases and injuries in 204 countries and territories, 1990–2019: a systematic analysis for the global burden of disease study 2019. Lancet. (2020) 396:1204–22. doi: 10.1016/S0140-6736(20)30925-9, 33069326 PMC7567026

[5] United Nations. Transforming our world: The 2030 agenda for sustainable development 2015. United Nations: Department of Economic and Social Affairs: Sustainable Development (2023).

[6] Zhang L Tong Z Han R Guo R Zang S Zhang X . Global, regional, and National Burdens of ischemic heart disease attributable to smoking from 1990 to 2019. J Am Heart Assoc. (2023) 12:e028193. doi: 10.1161/JAHA.122.028193, 36718860 PMC9973632

[7] Ping Y Hang Q Liya W. Early prediction of high-cost inpatients with ischemic heart disease using network analytics and machine learning. Expert Syst Appl. (2022) 210:118541. doi: 10.1016/j.eswa.2022.118541

[8] Sadeghi M Jamalian M Mehrabani-Zeinabad K. The burden of ischemic heart disease and the epidemiologic transition in the eastern Mediterranean region: 1990-2019. PLoS One. (2023) 18:e0290286. doi: 10.1371/journal.pone.0290286, 37669274 PMC10479892

[9] Montone RA Rinaldi R Bonanni A Severino A Pedicino D Crea F . Impact of air pollution on ischemic heart disease: evidence, mechanisms, clinical perspectives. Atherosclerosis. (2023) 366:22–31. doi: 10.1016/j.atherosclerosis.2023.01.013, 36696748

[10] Somboonsin P Vardoulakis S Canudas-Romo V. A comparative study of life-years lost attributable to air particulate matter in Asia-Pacific and European countries. Chemosphere. (2023) 338:139420. doi: 10.1016/j.chemosphere.2023.139420, 37419148

[11] Al-Kindi SG Brook RD Biswal S. Environmental determinants of cardiovascular disease: lessons learned from air pollution. Nat Rev Cardiol. (2020) 17:656–72. doi: 10.1038/s41569-020-0371-2, 32382149 PMC7492399

[12] Brauer M Casadei B Harrington RA Kovacs R Sliwa K. Taking a stand against air pollution-the impact on cardiovascular disease: a joint opinion from the world heart federation, American College of Cardiology, American Heart Association, and the European Society of Cardiology. Circulation. (2021) 143:e800–4. doi: 10.1161/CIRCULATIONAHA.120.052666, 33506685

[13] Wang L Wu X Du J Cao W Sun S. Global burden of ischemic heart disease attributable to ambient PM2.5 pollution from 1990 to 2017. Chemosphere. (2021) 263:128134. doi: 10.1016/j.chemosphere.2020.128134, 33297122

[14] Albadrani M. Socioeconomic disparities in mortality from indoor air pollution: a multi-country study. PLoS One. (2025) 20:e0317581. doi: 10.1371/journal.pone.0317581, 39820922 PMC11737656

[15] GBD 2021 Diseases and Injuries Collaborators. Global incidence, prevalence, years lived with disability (YLDs), disability-adjusted life-years (DALYs), and healthy life expectancy (HALE) for 371 diseases and injuries in 204 countries and territories and 811 subnational locations, 1990-2021: a systematic analysis for the global burden of disease study 2021. Lancet. (2024) 403:2133–61. doi: 10.1016/S0140-6736(24)00757-8, 38642570 PMC11122111

[16] GBD 2021 Risk Factors Collaborators. Global burden and strength of evidence for 88 risk factors in 204 countries and 811 subnational locations, 1990-2021: a systematic analysis for the global burden of disease study 2021. Lancet. (2024) 404:244. doi: 10.1016/s0140-6736(24)00933-4 PMC11120204 38762324

[17] Zhou X Ruan W Jie H Liu H Wang T Li J . Global trends in ischemic heart disease mortality from 1990 to 2021 and 2036 projections: insights from GBD 2021 data. Glob Heart. (2025) 20:92. doi: 10.5334/gh.1486, 41079058 PMC12513345

[18] GBD 2021 Causes of Death Collaborators. Global burden of 288 causes of death and life expectancy decomposition in 204 countries and territories and 811 subnational locations, 1990-2021: a systematic analysis for the global burden of disease study 2021. Lancet. (2024) 403:2100–32. doi: 10.1016/s0140-6736(24)00367-2 38582094 PMC11126520

[19] Li C Fu Y Liu S Yu H Yang X Zhang M . The global incidence and disability of eye injury: an analysis from the global burden of disease study 2019. EClinicalMedicine. (2023) 62:102134. doi: 10.1016/j.eclinm.2023.102134, 37599904 PMC10432781

[20] GBD 2019 Mental Disorders Collaborators. Global, regional, and national burden of 12 mental disorders in 204 countries and territories, 1990-2019: a systematic analysis for the global burden of disease study 2019. Lancet Psychiatry. (2022) 9:137–50. doi: 10.1016/S2215-0366(21)00395-3, 35026139 PMC8776563

[21] Sang S Chu C Zhang T Chen H Yang X. The global burden of disease attributable to ambient fine particulate matter in 204 countries and territories, 1990-2019: a systematic analysis of the global burden of disease study 2019. Ecotoxicol Environ Saf. (2022) 238:113588. doi: 10.1016/j.ecoenv.2022.113588, 35525115

[22] Tan J Zhu Z Wang X Yang B. Global burden and trends of musculoskeletal disorders in postmenopausal elderly women: a 1990-2021 analysis with projections to 2045. Arthritis Res Ther. (2025) 27:127. doi: 10.1186/s13075-025-03587-8, 40537855 PMC12178034

[23] GBD 2021 Forecasting Collaborators. Burden of disease scenarios for 204 countries and territories, 2022-2050: a forecasting analysis for the global burden of disease study 2021. Lancet. (2024) 403:2204–56. doi: 10.1016/S0140-6736(24)00685-8, 38762325 PMC11121021

[24] Chen Q Li T Ding H Huang G Du D Yang J. Age-period-cohort analysis of epidemiological trends in pelvic fracture in China from 1992 to 2021 and forecasts for 2046. Front Public Health. (2024) 12:1428068. doi: 10.3389/fpubh.2024.1428068, 39040861 PMC11260792

[25] Huang D Lai H Shi X Jiang J Zhu Z Peng J . Global temporal trends and projections of acute hepatitis E incidence among women of childbearing age: age-period-cohort analysis 2021. J Inf Secur. (2024) 89:106250. doi: 10.1016/j.jinf.2024.106250, 39181413

[26] Luo Z Shan S Cao J Zhou J Zhou L Jiang D . Temporal trends in cross-country inequalities of stroke and subtypes burden from 1990 to 2021: a secondary analysis of the global burden of disease study 2021. EClinicalMedicine. (2024) 76:102829. doi: 10.1016/j.eclinm.2024.102829, 39309727 PMC11415963

[27] Zeng Q Jiang D. Global trends of interstitial lung diseases from 1990 to 2019: an age-period-cohort study based on the global burden of disease study 2019, and projections until 2030. Front Med (Lausanne). (2023) 10:1141372. doi: 10.3389/fmed.2023.1141372, 37554509 PMC10404716

[28] Wu X Du J Li L. Bayesian age-period-cohort prediction of mortality of type 2 diabetic kidney disease in China: a modeling study. Front Endocrinol (Lausanne). (2021) 12:767263. doi: 10.3389/fendo.2021.767263, 34777260 PMC8586507

[29] You Y Wang Z Yin Z Bao Q Lei S Yu J . Global disease burden and its attributable risk factors of peripheral arterial disease. Sci Rep. (2023) 13:19898. doi: 10.1038/s41598-023-47028-5, 37963985 PMC10645774

[30] Yang X Fang Y Chen H Zhang T Yin X Man J . Global, regional and national burden of anxiety disorders from 1990 to 2019: results from the global burden of disease study 2019. Epidemiol Psychiatr Sci. (2021) 30:e36. doi: 10.1017/S2045796021000275, 33955350 PMC8157816

[31] Xue P Lin L Li P Cheng S Chen D Fan M . Global, regional, and national epidemiology of ischemic heart disease among individuals aged 55 and above from 1990 to 2021: a cross-sectional study. BMC Public Health. (2025) 25:985. doi: 10.1186/s12889-025-22193-6, 40075403 PMC11905664

[32] Lee KK Bing R Kiang J Bashir S. Adverse health effects associated with household air pollution: a systematic review, meta-analysis, and burden estimation study. Lancet Glob Health. (2020) 8:e1427–34. doi: 10.1016/S2214-109X(20)30343-0, 33069303 PMC7564377

[33] Ikaheimo TM. Cardiovascular diseases, cold exposure and exercise. Temperature (Austin). (2018) 5:123–46. doi: 10.1080/23328940.2017.1414014, 30377633 PMC6204981

[34] Irfan H. Air pollution and cardiovascular health in South Asia: a comprehensive review. Curr Probl Cardiol. (2024) 49:102199. doi: 10.1016/j.cpcardiol.2023.102199, 37977414

[35] Fleg JL Forman DE Berra K Bittner V. Secondary prevention of atherosclerotic cardiovascular disease in older adults: a scientific statement from the American Heart Association. Circulation. (2013) 128:2422–46. doi: 10.1161/01.cir.0000436752.99896.22, 24166575 PMC4171129

[36] Wang J Huang Y Feng N Xu L. Global disease burden attributable to high body mass index in young adults from 1990 to 2019, with projections to 2050: a systematic analysis for the global burden of disease study 2019. Diabetes Metab Res Rev. (2024) 40:e70007. doi: 10.1002/dmrr.70007, 39535472

[37] de Bont J Jaganathan S Dahlquist M Persson Å Stafoggia M Ljungman P. Ambient air pollution and cardiovascular diseases: an umbrella review of systematic reviews and meta-analyses. J Intern Med. (2022) 291:779–800. doi: 10.1111/joim.13467, 35138681 PMC9310863

[38] Safiri S Karamzad N Singh K. Burden of ischemic heart disease and its attributable risk factors in 204 countries and territories, 1990-2019. Eur J Prev Cardiol. (2022) 29:420–31. doi: 10.1093/eurjpc/zwab213, 34922374

[39] Tan J Xue M Li H Liu Y He Y Liu J . Global, regional, and National Burden of ischemic heart disease attributable to 25 risk factors and their summary exposure value across 204 countries with different socio-demographic index levels, 1990-2021: a systematic fixed-effects analysis and comparative study. Clin Epidemiol. (2025) 17:105–29. doi: 10.2147/CLEP.S510347, 39996156 PMC11849418

[40] Pega F Nafradi B Momen NC. Global, regional, and national burdens of ischemic heart disease and stroke attributable to exposure to long working hours for 194 countries, 2000-2016: a systematic analysis from the WHO/ILO joint estimates of the work-related burden of disease and injury. Environ Int. (2021) 154:106595. doi: 10.1016/j.envint.2021.106595, 34011457 PMC8204267

[41] Wang Y Li Q Bi L Wang B Lv T Zhang P. Global trends in the burden of ischemic heart disease based on the global burden of disease study 2021: the role of metabolic risk factors. BMC Public Health. (2025) 25:310. doi: 10.1186/s12889-025-21588-9, 39856644 PMC11763131

[42] Li J Lin S Pei H Xie G Pei L Chen G. Age-period-cohort analysis of cardiovascular disease trends in middle-aged and older adults: cross-country comparison across HRS, ELSA, SHARE, and CHARLS. J Glob Health. (2025) 15:04260. doi: 10.7189/jogh.15.04260, 40936419 PMC12434385

[43] GBD 2019 Risk Factors Collaborators. Global burden of 87 risk factors in 204 countries and territories, 1990-2019: a systematic analysis for the global burden of disease study 2019. Lancet. (2020) 396:1223–49. doi: 10.1016/S0140-6736(20)30752-2, 33069327 PMC7566194

[44] Bianco G Espinoza-Chavez RM Ashigbie PG. Projected impact of climate change on human health in low- and middle-income countries: a systematic review. BMJ Glob Health. (2024) 8:5550. doi: 10.1136/bmjgh-2024-015550, 39357915 PMC11733072

[45] Gao J Kovats S Vardoulakis S. Public health co-benefits of greenhouse gas emissions reduction: a systematic review. Sci Total Environ. (2018) 627:388–402. doi: 10.1016/j.scitotenv.2018.01.193, 29426161

[46] Huang J Zeng Q Pan X Guo X Li G. Projections of the effects of global warming on the disease burden of ischemic heart disease in the elderly in Tianjin, China. BMC Public Health. (2019) 19:1465. doi: 10.1186/s12889-019-7678-0, 31694683 PMC6836533

[47] Anand S Bradshaw C Prabhakaran D. Prevention and management of CVD in LMICs: why do ethnicity, culture, and context matter? BMC Med. (2020) 18:7. doi: 10.1186/s12916-019-1480-9, 31973762 PMC6979081

